# Identifying opportunities for global surgery in Cameroon: an analysis of existing health policies and events

**DOI:** 10.11604/pamj.2024.47.143.38399

**Published:** 2024-03-27

**Authors:** Berjo Dongmo Takoutsing, Geneviève Endalle, Wah Praise Senyuy, Bilong Mbangtang Celestin, Gaetan Konfo Kwasseu, Pride Bobga Tanyi, Desmond Tanko Jumbam, Ulrick Sidney Kanmounye

**Affiliations:** 1 Research Department, Association of Future African Neurosurgeons, Yaounde, Cameroon; 2 Faculty of Health Sciences, University of Buea, Buea, Cameroon; 3 Faculty of Medicine and Biomedical Sciences, University of Yaounde I, Yaounde, Cameroon; 4 Department of Policy and Advocacy, Operation Smile Ghana, Accra, Greater Accra, Ghana

**Keywords:** Cameroon, global health, global surgery, health policy, health system strengthening

## Abstract

**Introduction:**

the burden of diseases amenable to surgery, obstetrics, trauma, and anesthesia (SOTA) care is increasing globally but low- and middle-income countries are disproportionately affected. The Lancet Commission on Global Surgery proposed National Surgical, Obstetrics, and Anesthesia Plans as national policies to reduce the global SOTA burden. These plans are dependent on comprehensive stakeholder engagement and health policy analysis. Objective: in this study, we analyzed existing national health policies and events in Cameroon to identify opportunities for SOTA policies.

**Methods:**

we searched the Cameroonian Ministry of Health´s health policy database to identify past and current policies. Next, the policies were retrieved and screened for mentions of SOTA-related interventions using relevant keywords in French and English, and analyzed using the 'eight-fold path´ framework for public policy analysis.

**Results:**

we identified 136 policies and events and excluded 16 duplicates. The health policies and events included were implemented between 1967 and 2021. Fifty-nine policies and events (49.2%) mentioned SOTA care: governance (n=25), infrastructure (n=21), service delivery (n=11), workforce (n=11), information management (n=10), and funding (n=8). Most policies and events focused on maternal and neonatal health, followed by anesthesia, ophthalmologic surgery, and trauma. National, multinational civil society organizations and private stakeholders supported these policies and events, and the Cameroonian Ministry of Public Health was the largest funder.

**Conclusion:**

most Cameroonian SOTA-related policies and events focus on maternal and neonatal care, and health financing is the health system component with the least policies and events. Future SOTA policies should build on existing strengths while improving neglected areas, thus attaining shared global and national goals by 2030.

## Introduction

Global surgery aims to ensure access to timely, safe, and affordable surgery, obstetrics, trauma, and anesthesia (SOTA) care globally with a focus on low- and middle-income countries such as Cameroon [1,2]. In 2015, the seminal Lancet Commission on Global Surgery (LCoGS) report showed that about 5 billion people globally lack access to safe SOTA care, most of them in low- and middle-income countries [2]. That same year saw the publication of other landmark documents, including the Diseases Control Priorities (III) by the World Bank with a volume dedicated to surgical care and the passing of World Health Assembly resolution 68.15 by the World Health Organization explicitly recognizing surgical and anesthesia care as a part of Universal Health Care (UHC). Furthermore, following the recommendation of the LCoGS, National Surgical, Obstetric, and Anaesthesia Plans (NSOAPs) have been developed in many sub-Saharan African countries, including; Tanzania, Zambia, Madagascar, Ethiopia, Rwanda, and Nigeria as part of their national health policy strategy or plan [3]. NSOAPs are designed to strengthen surgical systems, covering every health system domain: infrastructure, surgical workforce, service delivery, information management, governance, and financing [4]. They aim to systematically and comprehensively improve access to high-quality, and affordable surgical care nationally.

Cameroonians face numerous barriers to accessing timely, affordable, and safe SOTA care. With an average catchment area of 28.25 km, the mean proportion of Cameroonians living 30 minutes, 1 hour, and 2 hours from a surgical facility is 74%, 88%, and 98% respectively [5]. Unfortunately, most of these facilities lack access to basic surgical amenities [6]. The mean national surgical specialist workforce density is about 1.15 per 100,000 population, significantly below the LCOGS target of at least 20 surgical specialists per 100,000 population [7]. Although national-level data is not available, a recent study found that 2,460 surgical procedures per 100,000 population are conducted by a handful of specialist surgeons in the Fako Division (South West, Cameroon) [8,9]. Furthermore, people in Cameroon with SOTA-related pathologies face significant financial hardship as a result of seeking SOTA care. According to the World Bank, the risks of catastrophic and impoverishing SOTA-care-related expenditures are estimated at 57% [10], and 39% respectively [11]. Maternal, neonatal, and under-five mortality remains high, estimated at 529, 2,600, and 7,500 per 100,000 live births respectively [12-14]. Mindful of these SOTA challenges, the Cameroonian government began developing an NSOAP under the leadership of the Ministry of Public Health (MINSANTE) and in collaboration with international civil society organizations [15].

In developing NSOAPs, existing health policies should be taken into account to avoid redundancy and conflicting policies [16]. Health policy research “seeks to understand and improve how societies organize themselves in achieving collective health goals, and how different actors interact in the policy and implementation processes to contribute to policy outcomes” [17]. To reach this goal, health policy analysis identifies opportunities for collaboration, pooling of resources, and synergy. Also, it informs the creation, costing, implementation, and strengthening of evidence-based policies. Some health policy analyses have been conducted in Cameroon, but to our knowledge, no study has focused specifically on assessing health policies related to SOTA care in Cameroon. Therefore, in this study, we aimed to analyze Cameroon's health policies and events (HP&Es) to identify opportunities for surgical system strengthening in Cameroon. We consider as health policy any document with a decision, plan, law, action, outlined priorities, and expected roles that are undertaken to achieve specific healthcare goals in Cameroon [18]. A health event is considered the occurrence of any event related to strengthening the delivery of care in Cameroon. The study findings could inform the ongoing NSOAP development and implementation, and as a result, may lead to the successful attainment of global surgery goals by 2030.

## Methods

This health policy analysis of past and current HP&Es in Cameroon was conducted using MINSANTE´s online health policy database [19].

**Search strategy:** after retrieving the HP&E from MINSANTE´s database, each policy document was reviewed by at least two of six authors (BDT, GE, PWS, GKK, CBM, and PBT) independently using variants and combinations of search terms related to SOTA-care in English and French. A combination and variation of the following search terms were used; “surgery, obstetric, trauma, anesthesia” (Annex 1, Annex 2). All HP&Es were retrieved from the MINSANTE online health policy database [19] from Cameroon´s Independence Day (January 1, 1960) to September 1, 2021. All the HP&Es retrieved from the search were exported to an Excel proforma sheet (Microsoft, Redmond, Washington, USA), identified, and duplicates deleted.

**Data extraction:** an Excel spreadsheet for data extraction was created, and the authors (BDT, GE, PWS, GKK, CBM, and PBT) participated in a pilot. The pilot consisted of independently extracting and categorizing data (year of health event or publication of the policy document, SOTA specialty, health system components involved, stakeholders involved, and funder) for each article. The pilot results were used to standardize data extraction and clear up ambiguities. Next, the authors completed data extraction for all included HP&Es. SOTA specialties were classified using the Association of American Medical Colleges classification [20]. At the same time, the national surgical system domains were divided into six groups (workforce, service delivery, information management, medical technologies and infrastructure, health financing, and leadership and governance) as per the LCoGS and WHO health systems building blocks [21]. Governance involved the Ministry of Health or local authorities establishing specific agendas and coordinating the smooth running of interventions established to address specific problems. These problems were identified by the information management arm which essentially gave feedback on specific SOTA-related national indicators (e.g maternal mortality rate, and factors contributing to its change). Infrastructure, consisted of donations of hospital equipment from national, multinational civil society organizations and private stakeholders. An HP&E was considered to be on the workforce if it was geared towards opening calls for the recruitment of SOTA-related personnel and training programs. Also, the finance component was considered if the HP&E involved generous monetary donations to health facilities and innovative health financing initiatives. Finally, service delivery was considered if it focused on the delivery of specific health care packages usually informed by the information management arm (e.g. provision of obstetric kits, refocused antenatal consultations, and caesarean section to improve maternal mortality) [22]. The data extractions were compared for discrepancies that were resolved between the authors. The senior author (USK) was consulted for arbitration when the authors could not reach a consensus.

**Figure 1:**
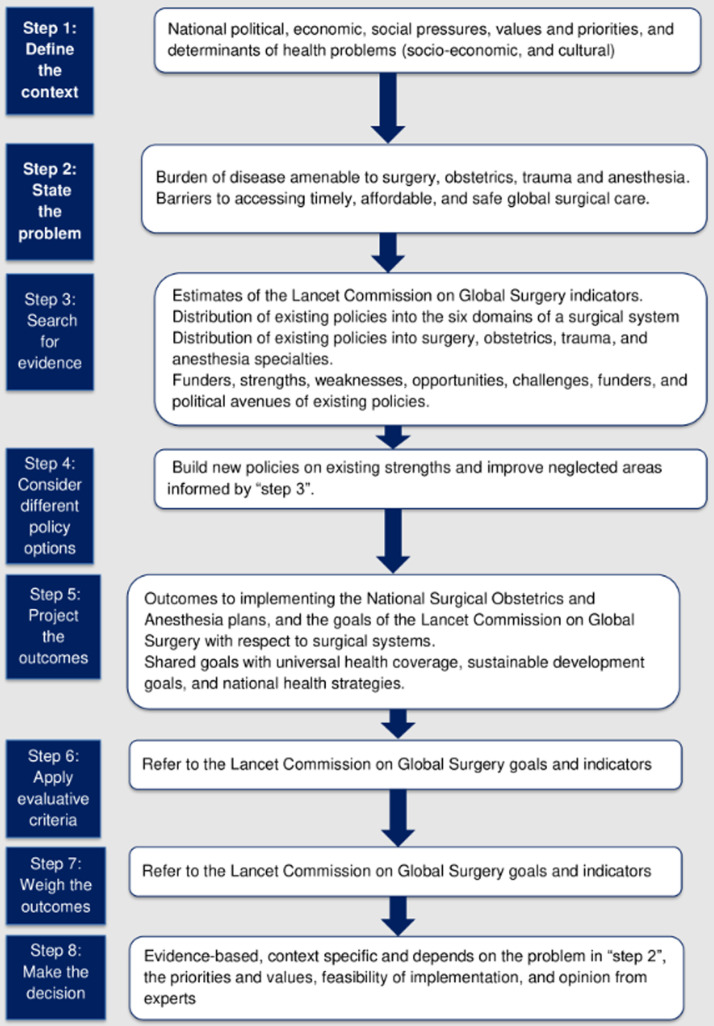
the eight-fold path analytical framework for global surgery-focused policy analysis

**Data analysis:** the relevant HP&Es documents were analyzed using the ‘eight-fold path’ framework for public policy analysis ((1) define the context; (2) state the problem; (3) search for evidence; (4) consider different policy options; (5) project the outcomes; (6) apply evaluative criteria; (7) weigh the outcomes; (8) make the decision) [23]. The analysis was done within a global surgery context (Figure 1), and the results were presented narratively.

## Results

**Figure 2:**
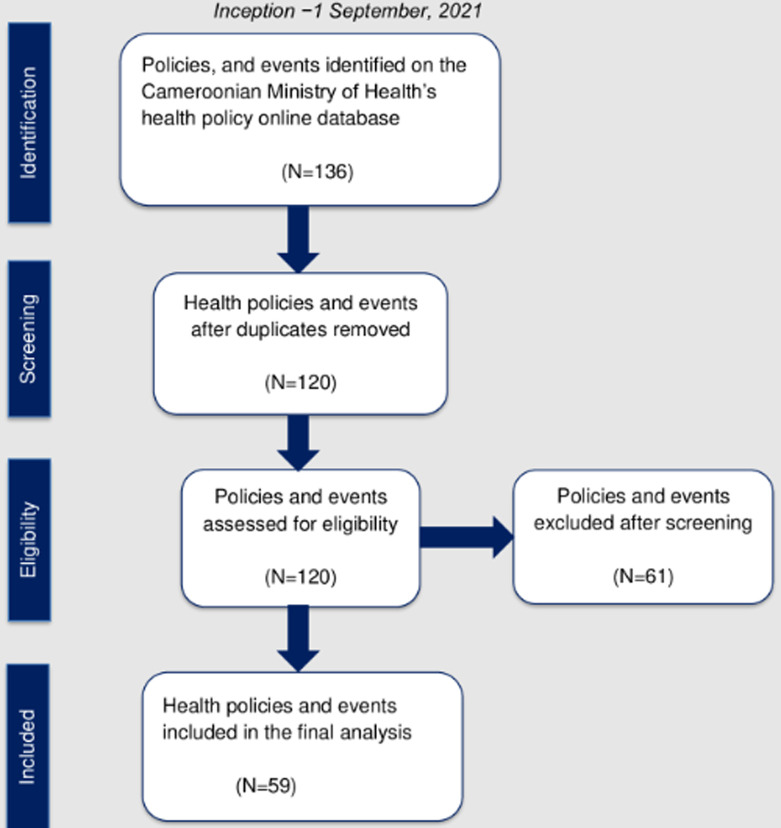
search, screening, and eligibility flow chart for the health policies and events included in the analysis

We identified 136 HP&Es in total and excluded 16 duplicates. Of note, 59 (49.2%) of the included HP&Es mentioned SOTA care at least once and were implemented between 1967, and 2021 (Figure 2). The majority of the HP&Es focused on obstetrics (n=41, 69.5%), followed by surgery (n=27, 45.8%), and anesthesia (n=19, 32.2%) (Table 1). The surgical HP&Es were focused primarily on ophthalmologic (n=11, 18.6%), gyneco-obstetrical (n=6, 10.2%), and maxillofacial surgeries (Table 1). Nineteen (32.2%) HP&Es focused on Bellwether surgical procedures (open fracture treatment, emergency laparotomy, and cesarean section) (Table 1).

**Table 1:** frequency of health policies and events on surgery, obstetrics, trauma, and anesthesia

Specialty	Number of health policies and events (percentage)
Obstetrics	41(69.5)
Overall surgery	27 (45.8)
Ophthalmologic surgery	11 (18.6)
Non-specific surgery*	9 (15.3)
Gyneco-Obstetrical surgery†‡	6 (10.2)
Maxillo-facial surgery	6 (10.2)
Ear-Nose and Throat surgery	3 (5.1)
Orthopaedic surgery‡	3 (5.1)
General surgery‡	3 (5.1)
Neurosurgery	2 (3.4)
Plastic and Reconstructive surgery	2 (3.4)
Urological surgery	1 (1.7)
Cardiac surgery	1 (1.7)
Anesthesia	19 (32.2)
Trauma‡	11 (18.6)

* : non-specific surgery represents one in which the precise surgical specialty could not be isolated; †: gyneco-obstetrical surgery stands for cesarian section (n=2, 3.4%), and obstetrical fistula surgery (n=4, 6.8%); ‡: nineteen (32.2%) health policies and events were on bellwether surgical procedures

From a health system perspective, the distribution of HP&Es was: governance (n=25, 42.4%), infrastructure (n=21, 35.6%), service delivery (n=11, 18.6%), workforce (n=11, 18.6%), information management (n=10, 17.0%), and health financing (n=8, 13.6%) (Table 2). Governance was the most common theme for obstetrics (n=20, 33.9%), surgery (n=11, 18.6%), trauma (n=7, 11.9%), and anesthesia-related (n=7, 11.9%) HP&Es (Table 2).

**Table 2:** distribution of health policies and events on surgery, obstetrics, trauma, and anesthesia into the six domains of a health system (n (%); N=59)

WHO health system domains	Surgery	Obstetrics	Trauma	Anesthesia	Total
Health financing	3 (5.1)	6 (10.2)	3 (5.1)	4 (6.8)	8 (13.6)
Information management	4 (6.8)	8 (13.6)	3 (5.1)	3 (5.1)	10 (16.9)
Workforce	7 (11.9)	8 (13.6)	3 (5.1)	5 (8.5)	11 (18.6)
Service delivery	9 (15.3)	6 (10.2)	5 (8.5)	6 (10.2)	11 (18.6)
Infrastructure	5 (8.5)	10 (16.9)	1 (1.7)	6 (10.2)	21 (35.6)
Governance	11 (18.6)	20 (33.9)	7 (11.9)	7 (11.9)	25 (42.4)

WHO: World Health Organization

MINSANTE funded the majority of the HP&Es (n=31; 49.2%), followed by the Islamic Development Bank (n=5; 8%), and the United Nations Population Fund (n=5; 8%) (Table 3).

**Table 3:** funders of global surgery-oriented health policies and events (n (%); N=59)

Funder	Number of health policies and events (percentage)
Cameroon Ministry of Health (MINSANTE)	31 (49.2)
The United Nations Population Fund (UNFPA)	5 (8.0)
Islamic Development Bank (IDB)	5 (8.0)
World Health Organization (WHO)	3 (4.8)
Mercy Ships Humanitarian Organization	2 (3.2)
Association Jeunesse Verte du Cameroun (AJVC)	1 (1.6)
Solafrica Organization	1 (1.6)
Egyptian Government	1 (1.6)
Indian Government	1 (1.6)
Cameroon National Association for Family Welfare (CAMNAFAW)	1 (1.6)
Cimenteries du Cameroun (CIMENCAM)	1 (1.6)
Association for Rare Diseases and Orphan and Handicap Diseases (ALMOHA)	1 (1.6)
Cameroonian neurosurgeons	1 (1.6)
Doctors Without Borders Humanitarian Organization	1 (1.6)
Africa eye foundation	1 (1.6)
“Orbis International” NGO	1 (1.6)
U.S Agency for International Development (USAID)	1 (1.6)
International Labour Organization (ILO)	1 (1.6)
United Nations (UN)	1 (1.6)
Gesellschaft für Internationale (GIZ)	1 (1.6)
Samuel Eto'o	1 (1.6)
United Nations International Children Emergency Funds (UNICEF)	1 (1.6)
MSC Foundation	1 (1.6)
Reproductive, Maternal and Child Health Trust Fund	1 (1.6)
L'agence Universitaire pour L'innovation (AUI)	1 (1.6)
UBIPHARM Foundation	1 (1.6)
United Nations Development Program (UNDP)	1 (1.6)
German Government	1 (1.6)
French Government	1 (1.6)
Turkish Government	1 (1.6)
Tunisian Government	1 (1.6)
Septentrion Media	1 (1.6)

## Discussion

This study sought to analyze Cameroon's MINSANTE´s HP&Es to identify existing opportunities for aligning efforts to strengthen surgical systems in Cameroon with previous and current policy efforts in Cameroon. We found that ophthalmologic, maxillofacial, and gyneco-obstetrical surgeries were the most popular SOTA specialties in MINSANTE´s HP&Es. Trauma was the most underrepresented surgical system component. Policies on Bellwether surgical procedures were not as common as other surgical procedures. In addition, governance was the most represented health system component, and health financing HP&Es were uncommon for surgery and obstetrics-related interventions. Furthermore, service delivery was underrepresented in maternal and neonatal care.

**Opportunities for global surgery in the Cameroon national health strategy:** the 2016-2027 Cameroon health sector strategy represents the single most important health policy document in Cameroon [22]. As of 2019, maternal, neonatal, and under-five mortality in Cameroon is still unacceptably high despite some progress over the past decades [12-14]. Most of the maternal deaths incurred are in emergency obstetric care (post-partum hemorrhage (45.5%), labour dystocia (22.3%), pre-eclampsia (10.6%), and post-partum infections (8.9%)) [22]. This is as a result of multiple factors including; low rates of cesarian sections (2.4% in 2014 compared to the minimum of 5% recommended by the WHO), home deliveries, issues of finance and access to health care, socio-cultural barriers, poor antenatal care coverage, and lack of resources in health facilities [22]. This thus highlights the role of SOTA in reducing the burden of maternal deaths. In line with this, the 2016-2027 Cameroon health sector strategy in specific objective 3.2 aims by 2027 to have attained comprehensive management of maternal, newborn, child, and adolescent health problems in at least 80% of health facilities, and one of the proposed ways to achieve this goal is to train medical officers in emergency obstetric and neonatal care [22]. As highlighted in this study, the majority of HP&Es geared towards maternal and neonatal health corroborate their importance in Cameroon. In Cameroon, communicable diseases (CDs) account for 41.1% of deaths as of 2013, and there has been a significant increase in mortality due to NCDs from 23.3% in 2013 to 38.0% in 2019 [24,25]. As of 2019, the top ten causes of death in Cameroon were CDs (neonatal disease, lower respiratory infections, HIV/AIDs, diarrheal diseases, and malaria), NCDs (stroke, ischemic heart disease, and diabetes mellitus), and road injuries, a majority of which can be managed by appropriate SOTA interventions [26,27].

Surgery is paramount to the establishment of a sustainable and resilient health system. Poor access to health care and delayed medical interventions negatively affect disease progression. As a result, benign pathologies when left unattended may end up needing an operative intervention or at least a surgical consult [2]. The epidemiologic transition from CDs to NCDs [24,25] will only add to the importance of SOTA care, increasing surgical needs. The 2016-2027 Cameroon health sector strategy in specific objective 3.3 forecasts ensuring the management of medical and surgical emergencies, according to standard operating procedures in at least 80% of health facilities by 2027 [22], which is similar to the goal of having access to timely essential surgery stipulated by the LCoGS.

Proximity to a SOTA care facility is a good indicator of access, and district hospitals tend to be the closest surgical facilities in sub-Saharan Africa [2,28]. Cameroon district hospitals are designed to offer emergency and essential SOTA procedures [29] and play an important role in universal access to health [30]. The 2016-2027 Cameroon health sector strategy aims to strengthen health facilities partly by training general practitioners in emergency obstetric surgery [22]. Undoubtedly, district hospitals will play an important role in the Cameroonian NSOAP [30].

Surgical care is delivered by specialist surgeons, general practitioners, and non-physician clinicians [29]. Yet, formal HP&Es on the role of task-shifting/-sharing within SOTA are not available. Maternal and neonatal care is a step ahead because of a global push to increase the proportion of births by skilled attendants. There is an opportunity for SOTA care to learn from this experience to ensure that quality SOTA care is provided by task-sharers.

**Global surgery and universal health coverage:** a minority of Cameroonians have health insurance [31,32]. Fortunately, the Cameroonian government has committed to attaining the goal of 100% protection against impoverishment and financial catastrophe from out-of-pocket payments [2,33]. As previously mentioned, the World Health Organization recognizes SOTA care as an essential constituent of the UHC [2,33]. Moreover, it also recognizes NSOAPs as reliable long-term, strategic plans to strengthen emergency and essential surgical services [2,33]. By implementing an NSOAP, the Cameroonian government will likely boost access to safe and affordable SOTA care for its population and get closer to meeting the UHC financial risk protection goal.

**Global surgery and the sustainable development goals:** Cameroon is a signatory of the sustainable development goals (SDGs) [34,35]. Integrating SOTA care via NSOAPs into national health agendas can help achieve eight of the 17 SDGs (i.e., 1, 3, 5, 8, 9, 10, 16, and 17) [35]. Sustainable development goals 1, which aims to end poverty in all forms [36], is impacted by financial hardships such as those created due to seeking access to quality health care exacerbating poverty. SDG 3 seeks to ensure healthy lives and promote well-being for all ages [36]. The burden of diseases amenable to SOTA care is unacceptably high in Cameroon, and most cases go unmet [29,37]. Also, a significant number of welfare losses are due to avoidable deaths, of which surgical conditions amount to a significant fraction of the economic losses [38]. Hence, by reducing the SOTA-related disease and financial burdens, NSOAPs will help attain the SDGs.

Sustainable Development Goals (SDG) 5 seeks to achieve gender equality and empower the female gender [36]. Preventable maternal mortality and morbidity must be eradicated for these to be achieved. Violence of all forms, including genital mutilation of the female gender, is still existent [39], making SOTA (trauma and gynecological surgery) care crucial to help these women. For example, access to surgical treatment is imperative to decrease the risk of infections, intra, and post-partum complications, and address the urological, reproductive sequelae, and cervical cancer which has a prevalence of 13.8% in Cameroon [40]. Despite multiple policies on maternal and neonatal health, only six were on gyneco-obstetrical surgeries. Global surgery also seeks equity in the SOTA workforce and education [2]. Overcoming female under-representation in SOTA specialties will increase the specialist workforce shortage [41,42].

**Globalizing the impact of policies on surgical care:** as we situate our findings within the broader context of global surgery policies, it is essential to consider prior reviews that offer insights into the challenges and opportunities faced by various nations. A similar study conducted in Ghana addressed the challenges and opportunities within the surgical care domain [43]. Their work emphasized the necessity for comprehensive and context-specific strategies. Our findings align with their emphasis on maternal and neonatal health in SOTA policies, showcasing a shared priority across various African nations, and echoing the broader trends observed in the global surgical landscape [44].

In addition to addressing the specific surgical components, it is essential to expand on the policy implications of SOTA care for non-surgical stakeholders in Cameroon's health ecosystem. Policymakers should recognize that the impact of SOTA care extends beyond surgical specialties and influences broader health outcomes [45]. Integrating SOTA care into national health policies should involve consultations with non-surgical stakeholders, including public health officials, community leaders, and representatives from health financing bodies. By promoting interdisciplinary collaboration and engaging non-surgical stakeholders, policies can be crafted to address the interconnected nature of healthcare delivery, ensuring a comprehensive and sustainable approach to improving health outcomes across the spectrum of surgical and non-surgical care [46].

**Underrepresentation of trauma in SOTA policies:** trauma, a significant global health burden, is conspicuously underrepresented in existing Surgery, Obstetrics, Trauma, and Anesthesia (SOTA) policies in Cameroon. This deviation from the comprehensive scope advocated by global surgical initiatives raises critical questions about the reasons behind this imbalance [47]. We suggest that this may be due to the different public health priorities, resource allocation challenges, and data availability and visibility [48].

To begin with, policies often reflect prevailing public health priorities, and in the context of Cameroon, a considerable emphasis on maternal and neonatal health is evident. Hence, maternal and neonatal care, given its impact on health indicators, may have taken precedence in policy formulation, inadvertently sidelining trauma. Similar studies highlight maternal health as a focal point in surgical system strengthening over trauma [43,49]. Secondly, we have the problem of resource allocations [50]. Limited healthcare resources may steer policy priorities towards areas with perceived higher disease burdens or those garnering more attention. Trauma, while globally significant, may not have received commensurate attention in resource allocation decisions. Finally, the availability and visibility of data play a pivotal role in shaping policy agendas. Trauma data, particularly in resource-limited settings, may be less robust compared to maternal and neonatal health data, potentially leading to its underrepresentation. A study in Ghana noted the challenges in obtaining accurate surgical data, influencing policy formulation [43].

To address the underrepresentation of trauma in SOTA policies in Cameroon, a multi-faceted approach is warranted. Policymakers should: advocate for the inclusion of trauma in global surgery initiatives and raise awareness about its impact on public health to garner policy attention; develop strategies to reallocate resources effectively, ensuring a balanced approach to addressing various components of the surgical system; strengthen trauma data collection systems to enhance the visibility of traumatic conditions within the national health landscape. The analyses of existing trauma registries have been done in Cameroon [51-54]. Despite not being representative of the nation´s entire trauma burden, stakeholders should use this to inform future policies on trauma care alongside the creation and analysis of more trauma registries. By addressing these considerations, policymakers can work towards a more inclusive and holistic SOTA policy framework, aligning with the goals of global surgery initiatives, and meeting the diverse healthcare needs of the population.

**Governance and health financing in SOTA care:** in bolstering the governance and health financing aspects of SOTA care in Cameroon, strategic interventions can further enhance the resilience and effectiveness of the surgical system. Governance enhancements should include the establishment of robust regulatory frameworks, clear lines of authority, and efficient coordination mechanisms. Drawing inspiration from successful models in other low- and middle-income countries, such as the governance reforms in Rwanda, Cameroon can institute policies that promote transparency, accountability, and stakeholder engagement [55]. Furthermore, the integration of SOTA care into the broader health financing landscape should involve innovative funding mechanisms, public-private partnerships, and targeted investment in infrastructure [56]. Hence, aligning financial resources with strategic priorities can lead to tangible improvements in surgical system strengthening.

**Strengths and limitations:** this study has several strengths. The policy tracing technique was developed based on findings from the MINSANTE online database to ensure retrieval of all policy documents. The ‘eight-fold path’ public policy framework was used for analysis due to its simplicity and thus it helped build a comprehensive understanding of SOTA-oriented HP&Es. This study is, to our knowledge, the first Cameroon-based health policy analysis case study conducted on SOTA care.

As for the limitations, only the MINSANTE database was used to generate policy documents leaving out potential data from other sources. However, to the best of our knowledge, only public policy influences decisions from governmental stakeholders hence our choice for this sole database. While we sought to review all relevant policies from MINSANTE by reviewing their database, it is possible that not all developed policies, those not accessible online, were included in our study; however, the identification of health policy documents dating back from the Independence Day of Cameroon makes the authors trust the reliability of this online database to inform decision making.

## Conclusion

This paper illustrates past, present, and future opportunities for SOTA care in Cameroon. The recognition of the importance of SOTA care in strengthening Cameroon's health system dates back to 1967 and continues to date but remains insufficient. We have shown that SOTA care and NSOAPs can co-exist with the 2016-2027 health sector strategy, UHC, and SDGs to strengthen the entire healthcare system. Scaling up global surgery services can accelerate progress to achieve these shared goals between the LCoGS, UHC, and SDGs.

### Recommendations

**To the Cameroon Ministry of Health:** 1) for future health policies geared toward SOTA to build on existing strengths and improve neglected areas identified in this study; 2) effective implementation of the Cameroonian NSOAP via timely monitoring and evaluation of the LCoGS indicators should ensure the attainment of global surgery goals by 2030.

**To countries that are planning NSOAPs:** to carry out a similar analysis before the NSOAP design process to identify strengths, weaknesses, opportunities, and threats. This baseline analysis should equally help track the effectiveness of NSOAP interventions once they are implemented.

**To health policy researchers:** 1) to study neglected global surgery disciplines within surgical systems such as; global cardiothoracic surgery, global otolaryngology-head and neck surgery, global neurosurgery, and global urology; 2) to study neglected health system components like financing of interventions aimed at improving surgical care availability, delivery, and affordability.

#### What is known about this topic

- *The goal of NSOAPs is to improve the surgical health system so as to improve the disproportionate burden of SOTA-related disorders in low- and middle-income nations;*
- *To avoid duplication and inconsistency of policies, existing health policies should be considered while designing NSOAPs;*
- *The ongoing development of the Cameroonian NSOAP, warrants a health policy analysis of past and existing global surgery-oriented policies and events*.

#### What this study adds

- *This analysis identified strengths, weaknesses, opportunities, challenges, and stakeholders for targeted SOTA interventions in Cameroon;*
- *The majority of global surgery-related health policies and events in Cameroon focused on maternal and neonatal health, and finance was the surgical system domain with the lowest representation;*
- *Cameroon SOTA-related health policies and events were funded by national, and international civil society organizations and private stakeholders*.

## Annex

**Annex 1**: search terms for each global surgery component in English (PDF - 56 KB)

**Annex 2**: search terms for each global surgery component in French (PDF - 57 KB)
